# A Practical Approach for the In Vitro Safety and Efficacy Assessment of an Anti-Ageing Cosmetic Cream Enriched with Functional Compounds

**DOI:** 10.3390/molecules26247592

**Published:** 2021-12-15

**Authors:** Nicola Zerbinati, Sabrina Sommatis, Cristina Maccario, Serena Di Francesco, Maria Chiara Capillo, Giulia Grimaldi, Raffaele Rauso, Martha Herrera, Pier Luca Bencini, Roberto Mocchi

**Affiliations:** 1 Department of Medicine and Surgery, University of Insubria, 21100 Varese, Italy; nicola.zerbinati@uninsubria.it; 2 UB-CARE S.r.l.-Spin-Off, University of Pavia, 27100 Pavia, Italy; sabrina.sommatis@ub-careitaly.it (S.S.);cristina.maccario@ub-careitaly.it (C.M.); serena.difrancesco@ub-careitaly.it (S.D.F.);mariachiara.capillo@ub-careitaly.it (M.C.C.); research@ub-careitaly.it (G.G.); 3 Maxillofacial Surgery Unit, Department of Medicine and Surgery, University of Campania “Luigi Vanvitelli”, 81100 Naples, Italy; raffaele.rauso@unicampania.it; 4 Centro Avanzado de Dermatologia y Laser, San Pedro Sula 21104, Honduras; marthaelizaherrera@gmail.com; 5 Istituto di Chirurgia e Laser-Chirurgia in Dermatologia (I.C.L.I.D.), 20121 Milan, Italy; pl.bencini@iclid.it

**Keywords:** in vitro, 3D model, skin irritation, collagen, elastin, MMP-1, anti-ageing, ceramides, cosmeceuticals, safety

## Abstract

(1) Background: Cosmeceuticals are topical products applied to human skin to prevent skin ageing and maintain a healthy skin appearance. Their effectiveness is closely linked to the compounds present in a final formulation. In this article, we propose a panel of in vitro tests to support the efficacy assessment of an anti-ageing cream enriched with functional compounds. (2) Methods: biocompatibility and the irritant effect were evaluated on reconstructed human epidermis (RHE) and corneal epithelium (HCE) 3D models. After a preliminary MTT assay, normal human dermal fibroblasts (NHDF) and keratinocytes (HaCaT) were used to evaluate the extracellular matrix (ECM) protein synthesis, and interleukin-6 (IL-6) and metalloproteinase-1 (MMP-1) production. (3) Results: data collected showed good biocompatibility and demonstrated the absence of the irritant effect in both 3D models. Therefore, we demonstrated a statistical increase in collagen and elastin productions in NHDF cells. In HaCaT cells, we highlighted an anti-inflammatory effect through a reduction in IL-6 levels in inflammatory stimulated conditions. Moreover, the reduction of MMP-1 production after UV-B radiation was demonstrated, showing significant photo-protection. (4) Conclusion: a multiple in vitro assays approach is proposed for the valid and practical assessment of the anti-ageing protection, anti-inflammatory and biocompatible claims that can be assigned to a cosmetic product containing functional compounds.

## 1. Introduction

Cosmetics are topical products applied on human skin with the aim of cleaning the skin, improving the skin’s appearance and preventing effects related to skin ageing. In recent years, cosmetology has emerged as a translational science including such different research areas as medicine, chemistry, physics, microbiology and dermatology. The challenge of the current trend is the formulation of functional cosmetics with a more complex product process based on innovative and effective raw materials to raise the level of safety and biocompatibility [1]. The safety of cosmetic and personal care products is fundamental for manufacturers, importers, retailers and consumers. Recently, awareness of animal testing and the consequent “cruelty-free” topic has been a significant drive for the in vitro, ex vivo and in silico testing industry, promoting advancements and innovations to make them more useful and cost-effective. Furthermore, one of the challenges on this topic is the assessment of models that are similar to and representative of human skin.

Skin is the first protective barrier of the body; it protects against extrinsic factors such as electromagnetic radiation, pollution, infections, and external chemical and physical agents. The skin is composed of three major layers, with the most superficial being the epidermis, followed by the dermis and the deeper hypodermis. The epidermis consists of several layers: the stratum basale (SB), stratum spinosum (SP), stratum granulosum (SG) and stratum corneum (SC). The epidermis is a keratinized, stratified squamous epithelium characterized by several types of cells, such as Langerhans cells, Merkel cells, melanocytes, lymphocytes and the most predominant cells, keratinocytes [2]. Keratinocytes are highly specialized cells in constant transition from the deeper proliferative SB to the superficial and differentiated SC; during this process, they morphologically change into highly keratinized and nucleus-free squamous cells, then begin to produce keratin, cytokines, growth factors and complement factors. In the healthy epidermis, the proliferation of the stratum basale and the desquamation process of the stratum corneum is deeply regulated and produces the epidermis, the first defensive barrier with a key role in maintaining skin health. Deeper than the epidermis, the dermis and the hypodermis are composed of three major types of cells-fibroblasts, macrophages, and adipocytes. They contain blood and lymph vessels, nerves and connective tissue that includes collagen and elastin fibers that provide strength and elasticity to the skin [3]. Collagen is one of the most important proteins in vertebrates and represents one-third of the total protein of the human body. Its precursor, procollagen, is synthesized by dermal fibroblasts. Different types of collagen have been identified, but collagen type I is the most important. It constitutes 90% of the collagen present in our organism and is located especially in the skin, where it is the main structural component of the extracellular matrix (ECM) of the dermis. In the dermal matrix, collagen type III is also present, forming fibers arranged parallel to the skin surface that gives it strength and endurance. We also find collagen type IV that provides support to the tissues. With age, there is a progressive decrease in collagen production associated with an increase in its degradation [4]. Elastin is another structural protein, with a role closely linked with collagen. The decline in the amount of collagen and elastin in tissues is associated with ageing, weakening tissue integrity and strength [5]. The ageing process can be classified into two categories: chronological (or intrinsic) ageing, which is the result of a loss of soft tissue volume and is characterized by fine wrinkles and a thinning epidermis, and extrinsic ageing, which is predominantly caused by chronic sun exposure and characterized by deep wrinkles and hyperpigmentation. Skin ageing results in progressive atrophy of the dermis due to many intracellular changes and quantitative and structural modifications in collagen fibers that, in contrast to young skin, are fragmented and coarsely distributed. Aberrant collagen homeostasis is closely related to matrix metalloproteinase (MMP) activity; MMPs are a family of ubiquitous endopeptidases that can degrade ECM proteins. Physiologically, MMPs are finely regulated by the specific endogenous tissue inhibitors of metalloproteinases (TIMPs) that in intrinsic or extrinsic ageing are significantly reduced. This imbalance is the major cause of a progressive collagen fragmentation in the dermis, and consequently accelerates skin ageing. Reactive oxygen species (ROS) are one of the main causes of increased MMPs; in the skin. ROS are generated from different sources, such as ultraviolet irradiation. ROS production induces the mitogen-activated protein kinase (MAPK) family, leading to the activation of the transcription factor activator protein 1 (APatt-1) but also the inhibition of transforming growth factor (TGF)-β signaling, both of which are involved in the transcriptional regulation of several MMPs [6,7]. Many of these intracellular processes can be counteract using products containing compounds both from natural and synthetic sources that are able to maintain integrity, prevent the penetration of microorganisms, and avoid free radical formation and dehydration of several tissues [8]. In a cosmeceutical formulation, active ingredients are necessary to promote skin benefits. They should be able to penetrate skin barriers, act selectively in a target skin zone and overcome a careful scientific study of effectiveness. The primary requisite for a compound used in cosmetics is skin biocompatibility associated with the absence of any immunological response [9]. Then, it is important to highlight the effectiveness. In this overview, we created a panel of in vitro tests to support the efficacy assessment of an anti-ageing cosmetic cream enriched with functional compounds. Therefore, we focus our attention on the Ceramide Shield Cream provided by Matex Lab (Brindisi, Italy). Starting from accurate scientific research related to the functional compounds present in the formulation, the safety and the efficacy for anti-ageing purposes on monolayer cell lines and on reconstructed 3D models are the main salient claims on which our study has been focused.

## 2. Results

### 2.1. Evaluation of Cell Viability (MTT)

**Figure 1:**
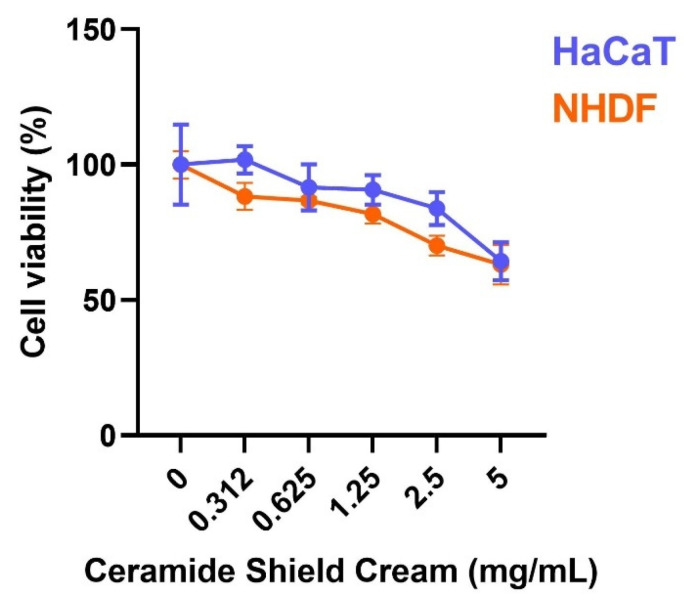
Cell viability (%) of HaCaT cells (in blue) and NHDF cells (in orange) after treatment with different concentrations of the cream (range between 0.312 and 5 mg/mL) with respect to untreated cells (*n* = 2; replicates = 3).

A cytotoxicity test is required to evaluate the effect of the cream on cell viability and to determine the appropriate concentrations required for the following assays. The product shows cytotoxic activity only after treatment with the highest tested concentrations of 2.5 and 5 mg/mL on human fibroblasts (NHDF cell line) after 24 h of treatment (range tested, 0.312–5 mg/mL). The same test was performed on human keratinocytes (HaCaT cell line) by dissolving the product in culture medium with 0.5% concentration of fetal bovine serum (FBS) to choose 1.25 and 2.5 mg/mL as the first concentrations with a viability ≥80% (Figure 1).

### 2.2. Collagen and Elastin Synthesis

**Figure 2:**
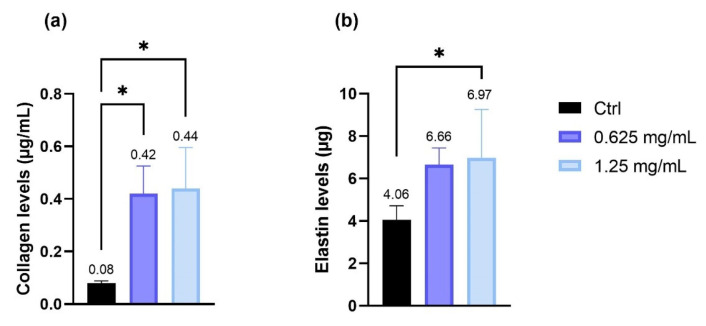
(**a**) Collagen levels and (**b**) elastin levels expressed as concentration (µg/mL) after treatment with the tested cream (0.625 and 1.25 mg/mL) in NHDF cells. * *p* values ≤ 0.05 were considered to be statistically significant compared with untreated cells (*n* = 3; replicates = 2).

To evaluate the possible modulation of collagen and elastin levels, NHDF cells were treated with the 2 concentrations (0.625 and 1.25 mg/mL) of the product which were shown to be non-cytotoxic from the preliminary MTT test. The obtained data highlight that treatment with both the tested concentrations induces an increase in collagen and elastin production compared to the control (Ctrl, untreated cells). The data were statistically significant (elastin production only after the treatment with 1.25 mg/mL), demonstrating a propensity of the product to modulate the production of the 2 structural proteins (Figure 2).

### 2.3. Antinflammatory Activity

**Figure 3:**
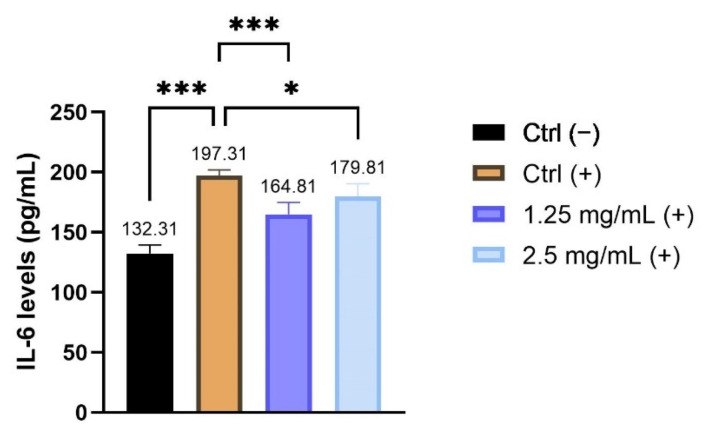
Quantitative analysis of IL-6 levels (pg/mL) after treatment with the cosmetic cream at the concentrations of 1.25 and 2.5 mg/mL and stimulation with TNF-α; Ctrl (−): untreated cells; Ctrl (+): cells treated only with TNF-α. * *p* values ≤0.05 and *** *p* values ≤ 0.001 were considered to be statistically significant compared with respective controls (*n* = 2; replicates = 2).

The possible soothing effect exerted by the enriched cream was evaluated after stimulation with TNF-α in HaCaT cells by quantifying the expression of IL-6 by the ELISA technique. The IL-6 levels were expressed as concentration in pg/mL in HaCaT cells treated with the tested product at the non-cytotoxic concentrations of 1.25 and 2.5 mg/mL compared to the positive control Ctrl (+) cells treated only with TNF-α (Figure 3). The reported data highlighted that treatment with the cream significantly reduces the release of IL-6 in human keratinocytes under inflammatory conditions. In particular, the inhibition percentage of interleukin release is equal to 13,62% and 8,87% after treatment with 1.25 and 2.5 mg/mL, respectively, indicating a highly significant modulation of the inflammatory cytokine exerted by the product.

### 2.4. Evaluation of MMP-1 Production

**Figure 4:**
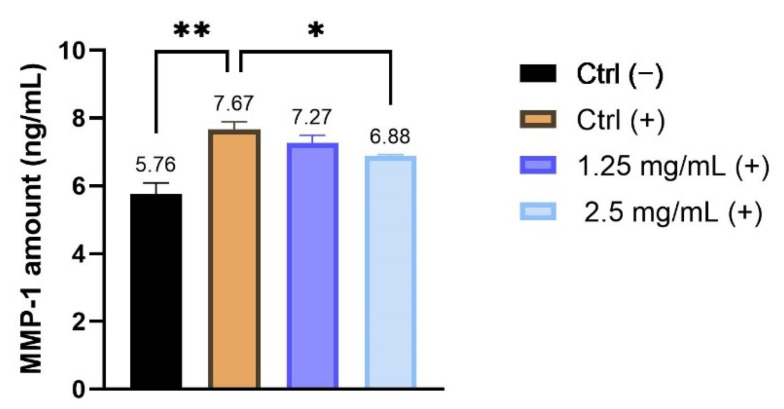
Quantitative analysis of MMP-1 levels (ng/mL) after treatment with the cosmetic cream at the concentrations of 1.25 and 2.5 mg/mL. Ctrl (−): untreated cells; UVB (+): irradiated cells. * *p* values ≤0.05 and ** *p* values ≤ 0.01 were considered to be statistically significant compared with irradiated cells (*n* = 2; replicates = 2).

The ability of the product to prevent photo-ageing was investigated in HaCaT cells by measuring their MMP-1 levels after 24 h treatment with the concentrations of 1.25 and 2.5 mg/mL selected by a preliminary MTT test and after irradiation with UVB. As shown in Figure 4, the results demonstrate that the UVB irradiation, UVB (+), markedly increases MMP-1 production by 24.89% (** *p* ≤ 0.01) compared to non-irradiated control cells, Ctrl (−). In the UVB condition, treatment with the 2 selected concentrations of the formulation induces a modest reduction of MMP-1 levels after 24 h compared to the positive control cells which were UVB-irradiated, UVB (+). In particular, it is possible to observe a reduction of 5.15% and 10.3% after treatment with 1.25 and 2.5 mg/mL, respectively (* *p* ≤0.05 after treatment with 2.5 mg/mL).

### 2.5. Skin Irritation on the RHE Model

**Figure 5:**
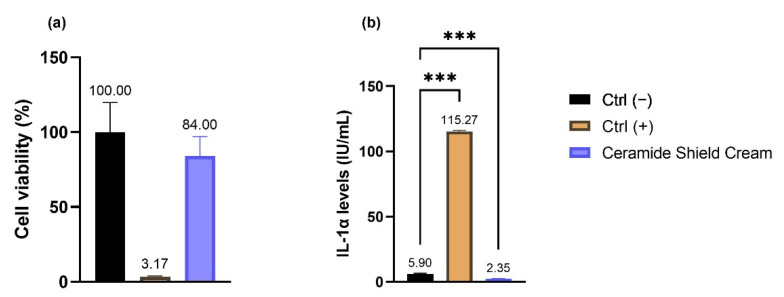
(**a**) Cell viability expressed as a percentage (%) of RHE treated with Ceramide Shield Cream. Ctrl (−): RHE treated with DPBS; Ctrl (+): RHE treated with SDS as the irritating stimulus. (**b**) IL-1α amount (IU/mL) in the medium after treatment with Ceramide Shield Cream; Ctrl (−): cells treated with DPBS; Ctrl (+): RHE treated with SDS. Values of *** *p* ≤ 0.001 were considered statistically significant compared with Ctrl (−) by one-way ANOVA statistical analysis followed by Fisher’s LSD test.

To evaluate the potential skin irritation risk of the enriched cream, cell viability by MTT assay and IL-1α amount analyzed by ELISA kit were investigated on 3D reconstructed human epidermis (RHE). The results obtained on the RHE 3D model (Figure 5) demonstrate the absence of a potential cream-related risk of irritation (viability of 84% and an amount of interleukin IL-1α released of 2.35 IU/mL).

### 2.6. Eye Irritation on the HCE Model

**Figure 6:**
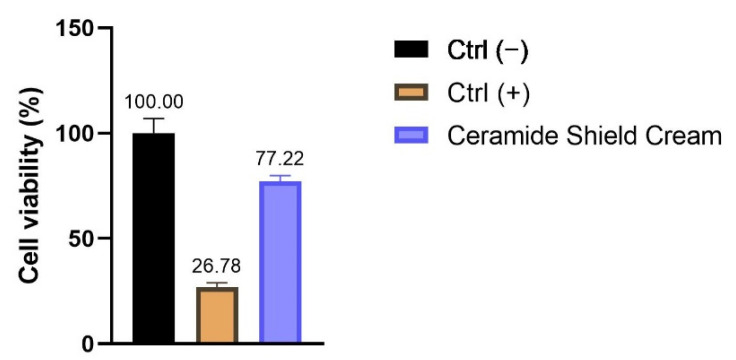
Cell viability expressed as a percentage (%) of HCE treated with Ceramide Shield Cream. Ctrl (−): HCE treated with DPBS; Ctrl (+): HCE treated with SDS as the irritating stimulus.

To evaluate the potential eye irritation risk of the enriched cream, cell viability was investigated on 3D human corneal epithelium (HCE) by MTT assay and according to the DB-ALM n°190 [10]. The results obtained demonstrate the absence of a potential cream-related risk or irritation (viability of 77%), as shown in Figure 6.

## 3. Discussion

In human anatomy, skin is the integument that provides protection and receives sensory stimuli from the external environment. The skin architecture presents three layers of tissue: the epidermis, which contains the primary protective structure; the dermis, a fibrous layer that provides strength to the epidermis for the association between collagen fibers and glycosaminoglycans (GAGs); and the subcutis, a subcutaneous layer that supplies nutrients to the other upper layers and plays a key role in thermal body isolation [11]. Each layer is represented by a peculiar cellular and tissue organization. The epidermis is classified as epithelial tissue composed of stratified squamous epithelia containing several cell types, including keratinocytes, melanocytes, Langerhans cells and Merkel cells. The dermis connects the epidermis to the hypodermis; it provides structure and elasticity due to the high content of collagen and elastin, two of the most abundant structural proteins in the human body. The dermis is composed of different types of connective tissues, including areolar and dense irregular connective tissue; fibroblasts are the most representative cellular population in this layer. The hypodermis plays a key role for the storage of fat (energy storage). It provides the link between the upper skin layers (dermis and epidermis) and underlying tissues (bones and cartilage), and it supports body temperature regulation. The hypodermis contains a varied cellular population such as fibroblasts, adipose cells, and macrophages [2].

Cosmetics are products used for cleansing or to protect the skin from exogenous and endogenous harmful factors. The major claims for a skin care formulation are represented by its soothing effect, its photoprotection as well as its ability to improve skin integrity and elasticity, enhancing epithelium texture and conferring a healthy and young appearance to the skin. The effectiveness of a skin care product is closely linked to its formulation, which usually can include synthetic or natural ingredients. In 1961, Raymond Reed, for the first time, coined the term “cosmeceuticals”, which was designed for these formulations that contain active ingredients conferring a benefit to the skin by penetrating the skin barrier and eliciting a specific mechanism of action in a targeted biological process [12].

From 11 July 2013, article 18 of Regulation (EC) No. 1223/2009 reaffirms what was already stated in Directive 76/768/EEC and subsequent amendments. It introduced for the first time specific provisions for the gradual elimination of tests on animals that were “conducted to comply with the provisions of that directive; that is, conducted for the study of the toxicity of cosmetic ingredients and products in order to evaluate their safety for human health“[13]. Consequently, in recent years, the set-up of faster and cheaper in vitro methods for the assessment of the efficacy of final skin care formulations as well as raw materials are of primary interest. In several research works, keratinocyte and fibroblast cell lines have been used to design a panel of in vitro methods to demonstrate the efficacy of raw materials capable of exerting an effect on key cellular mechanisms, such as proliferation, inflammation, oxidative damage and DNA repair. The results obtained from these studies have proved to be a valid screening of efficacy for the selection of these bioactive raw materials in final skin care formulations [14,15]. In our study, we collected bibliographic information about the main functional ingredients present in the formulation to be tested, and we designed a tailored panel of in vitro tests. The skin care product “Ceramide Shield Cream” provided by Matex Lab S.p.a. (Brindisi, Italy) was analyzed because of the presence of many functional compounds. Specifically, ceramides restore the hydrolipid barrier, replenish the stratum corneum, protect from harmful external injury, relieve irritation, and protect against dehydration.

Their general formula is CH_3_(CH_2_)XACHOHCH(NH_2_)CH_2_OH, where “A” represents the double bond -CH=CH- in sphingosine, -CH_2_-CHOH- in phytosphingosine and -CH2-CH2- in dihydrosphingosine, while the “X” value is between 10 and 16. Seven different types of ceramides have been found in human epidermis, and they differ from each other in fatty acid chain length. Problems may arise if there is a coupling between ceramides and phytosphingosine (PS) in the cosmetic formulation, because it would make the compound extremely insoluble: this problem is avoided in the product by using glycerol derivatives to solubilize ceramides into the ester base [16]. Ceramides and PS are known to have a positive effect on ECM remodeling via their inhibition of the transcription factor activator protein-1 (AP-1), which leads to an increase in procollagen-I and a reduction in the MMP-1 expression by adult human fibroblasts [17]. The probiotic peptide (Heptapeptide-4/10) improves the skin’s defense system by promoting the balance of the microbiota. It improves skin cell cohesion and strengthens vulnerable skin, making it more resilient. *Centella asiatica* is another important natural bioactive substance rich in flavonoids, phenolic acids, triterpenoids, amino acids, vitamins and sugars. Its extract stimulates skin regeneration and presents remarkable antioxidant, anti-inflammatory and antimicrobial effects that make this substance useful in dermatology for the treatment of burns, wounds, psoriasis and scleroderma. This natural substance can stimulate fibroblast proliferation and an increase in type I collagen production caused by the activation of the small mothers against decapentaplegic (SMAD) signaling pathway [18]. Other investigations suggest that its anti-ageing molecular mechanism could be associated with its ability to interfere with the gene expression of MMPs, cytosolic Cu/Zn-superoxide dismutase (SOD1) and mitochondrial Mn-superoxide dismutase (SOD2) [19]. Starting from these topics, our study was firstly focused on the cytotoxicity and the possible irritant effect of the skin care cream. The results show cytotoxic activity only after treatment with the highest tested concentrations on human fibroblasts (NHDF cell line) and keratinocytes (HaCaT cell line). The potential risks of skin and eye irritation were evaluated on reconstructed human epidermis (RHE) and human corneal epithelium (HCE) 3D models following the reference standard methods [10,20]. The results show a viability of 84% and an amount of interleukin IL-1α less than 9 IU/mL (the threshold to identify a substance as irritant) on RHE and a viability of 77% in HCE, confirming the biocompatibility of the skin care cream. The two concentrations (0.625 and 1.25 mg/mL) of the product were shown to be non-cytotoxic from the MTT test on NHDF cells, which were selected to assess collagen and elastin stimulation, and the results show a significant increase in both proteins involved in extracellular matrix (ECM) organization, demonstrating a rejuvenating effect of the dermocosmetic. To verify its soothing effect and anti-photoaging activity, the cytotoxicity of the tested cream was evaluated on HaCaT cells, and the two concentrations which were shown to be non-cytotoxic from the MTT test (1.25 and 2.5 mg/mL) were selected to quantify the IL-6 levels after stimulation with a proinflammatory stimulus (tumor necrosis factor-alpha, TNF-α) and matrix metalloproteinase (MMP)-1 by a colorimetric ELISA kit.

IL-6 is a cytokine which is very important in the inflammatory response. It represents one of the major groups of acute-phase proteins and promotes inflammation in a chronic state [21]. IL-6 levels are increased in most inflammatory conditions, and they are often recognized as therapeutic targets. Therefore, in vitro models for the cytokine’s quantification in a keratinocyte cell line can be a useful and a sensitive way to evaluate the anti-inflammatory effect of a cosmetic product. The results obtained show a significant decrease in the IL-6 amount at both tested concentrations (1.25 and 2.5 mg/mL), demonstrating an anti-inflammatory activity of the functional cream.

MMPs are a complex family of enzymes able to regulate and remodel ECM, promoting the turnover of various proteins such as collagen. Among all the MMPs, the collagenase MMP-1 is responsible for the degradation of fibrillar collagen type I, the most abundant structural protein in the dermis. Thus, alterations in the expression of MMP-1 are scientifically correlated to skin photo-ageing. The ability to reduce the MMP-1 production in keratinocytes (HaCaT) after ultraviolet (UV)-B radiation was evaluated, showing a significative photo-protection at the higher tested concentration of 2.5 mg/mL.

Overall, our study shows multiple approaches for the efficacy assessment of a “cosmeceutical” formulation enriched with active ingredients; the integration of different in vitro models purposefully built based on the features of the formulation allows the attribution of anti-ageing protection, anti-inflammatory and biocompatible claims to the object of the investigation, the Ceramide Shield Cream.

## 4. Materials and Methods

### 4.1. International Nomenclature of Cosmetic Ingredients (INCI)

The functional classification of ingredients contained in the tested enriched cream is summarized in Table 1.

### 4.2. Cell Cultures

Two different monolayer cell lines and two 3D models were used to perform the assays. The cell lines were the human cell line of keratinocytes (HaCaT, BS code CL 168), provided by I.Z.L.E.R. (Zooprofilattico Institute of Lombardy and Emilia Romagna, Brescia, Italy) and the normal human dermal fibroblasts cell line (NHDF-Ad-Der Fibroblasts, code CC-2511, Lonza, Basel, Switzerland). The two 3D models were the reconstructed human epidermis (RHE) and human corneal epithelium 3D models (Episkin Laboratories, Lyon, France). HaCaT cells were grown in a complete medium constituted of Dulbecco’s modified Eagle’s medium (DMEM; Biowest, Nuaillé, Francia) supplemented with fetal bovine serum (FBS; Gibco-Fisher Scientific, Waltham, MA, USA), 1 mM L-glutamine (Capricorn Scientific, Ebsdorfergrund, Germany), and antibiotics (100 U/mL penicillin and 100 µg/mL streptomycin; Capricorn Scientific, Ebsdorfergrund, Germany). NHDF cells were grown in FGM-2 SingleQuot kit Supplement & Growth Factors (LONZA, Basilea, Switzerland) supplemented with the Detach kit (Promocell, Sickingenstr, Heidelberg, Germany). Both cell lines were grown in conditions of complete sterility and maintained in a humidified atmosphere incubation at 37 °C with an atmosphere of 5% carbon dioxide (CO_2_). For RHE and HCE models, two different media were used for maintenance and growth (Episkin Laboratories, Lyon, France).

### 4.3. Cell Viability via the MTT Assay

3-(4,5-dimethylthiazol-2-yl)-2,5-diphenyl tetrazolium bromide) (MTT) is a tetrazolium salt used to test cell proliferation and viability based on mitochondrial efficiency [22]. For the preparation of the assay, cells were seeded (1.5 × 10^4^ and 1.7 × 10^4^ for HaCaT and NHDF experiments, respectively) into 96-well plates. After 24 h, cells were treated with the tested product at 20 mg/mL concentration and following (1:2) dilutions (tested range 0.156–20 mg/mL) were dissolved and vortexed in complete colture medium; untreated cells were used as control. After 24 h of treatment, the culture medium was discarded and the cells were incubated with the MTT (SIGMA-ALDRICH, St. Louis, MO, USA) solution (1 mg/mL) at 37 °C for 2 h. Then, the solution was removed and replaced with 100 µL of dimethyl sulfoxide (DMSO, Honeywell, NC, USA). The absorbance was read at a wavelength of 570 nm using a microplate reader (Multiskan, Thermo Scientific, Waltham, MA, USA). Cell survival was calculated by measuring the difference in optical density (OD) of the tested product with respect to the control (untreated cells) (*n* = 2; replicates = 3) [23].

Cell viability (%) = [OD_570_ nm test product/OD5_70_ nm control] × 100 (1)

### 4.4. Evaluation of Collagen Synthesis in NHDF Cells

The ability of the product to modulate collagen production was evaluated in NHDF cells after 24 h treatment using a specific colorimetric kit (Sircol, Soluble Collagen Assay Kit, Biocolor Life Science, Carrickfergus, United Kingdom). NHDF cells were homogeneously seeded in a 24-well plate at a density of 8 × 10^4^ cells/well. After 48 h, 2 of the tested product concentrations proven to be non-cytotoxic and with the best solubility in the medium were chosen. Untreated cells were used as control. At the end of incubation, 200 μL of Tris-HCl, pH 7.4, containing polyethylene glycol, was added to the recovered supernatant for the isolation and concentration of collagen, then stored overnight at 4 °C. The day after, the measurement of collagen synthesis was performed according to the manufacturer’s instructions. A total of 200 μL of samples were then transferred into a 96-well plate for spectrophotometric OD readings at a wavelength of 555 nm. The quantification of the collagen amount in each sample was obtained by plotting the mean absorbance with the linear regression standard curve (0–10 μg/mL) (*n* = 3; replicates = 2).

### 4.5. Evaluation of Elastin Synthesis in NHDF Cells

Total elastin was evaluated in human fibroblasts after 24 h treatment using a colorimetric kit (FastinTM, Elastin Assay kit, Biocolor Life Science Assays, Carrickfergus, United Kingdom). This assay is a quantitative dye-binding method able to detect elastin as α-elastin (soluble tropoelastins, lathyrogenic elastins and insoluble elastins). For the preparation of the assay, cells were homogeneously seeded in a 12-well plate at 1.8 × 10^5^ and incubated at 37 °C in 5% CO_2_ humidified atmosphere. After 48 h, the same concentrations chosen for the collagen synthesis test were used. Untreated cells were used as control. At the end of treatment, cells were harvested and the quantification of the elastin amount in each sample was carried out following the manufacturer’s instructions. The quantification of the elastin amount in each sample was obtained by plotting the mean absorbance with the linear regression standard curve (0–50 μg) (*n* = 3; replicates = 2).

### 4.6. Evaluation of the Soothing Effect in HaCaT Cells

The soothing effect of a cosmetic product should result in the decrease of pro-inflammatory biometric parameters. Interleukin-6 (IL-6) is one of the most well-known inflammatory markers; it plays a key role in host defense mechanisms, immune response, acute phase reactions and keratinocyte activation [24]. The content of IL-6 after treatment with the tested product, Ceramide Shield Cream, was evaluated in HaCaT cells after 24 h of treatment using an enzyme-linked immunosorbent assay (ELISA) kit (Thermo Fisher, Waltham, MA, USA). For the preparation of the assay, cells were homogeneously seeded into a 96-well plate at a density of 1.5 × 10^4^ and incubated at 37 °C with a 5% CO_2_ humidified atmosphere. The following day, cells were treated with the product at the concentrations determined by the MTT test at 0.5% FBS (1.25 and 2.5 mg/mL); cells were stimulated with tumor necrosis factor-α (TNF-α; SIGMA-ALDRICH, St. Louis, MO, USA) during the last 6 h of treatment. At the end of treatment, supernatants were collected and used for coating on a specifically pretreated 96-well ELISA plate provided by the kit. The IL-6 standard supplied by the kit was reconstituted with ultrapure water and used to build the standard curve (15.6–1000 pg/mL). Samples, blanks and standard points were added to each well in duplicate, and the assay was performed according to the manufacturer’s instructions. The absorbance was then read at 450 nm using a microplate reader. Data were analyzed as mean ± standard deviation (SD) and the quantification (pg/mL of IL-6/µg protein) was obtained by plotting the mean data with a four-parameter logistic (4PL) standard curve representing the best fit. The modulatory activity was evaluated in terms of percentage of inhibition obtained by comparison of the mean values of the control and treated conditions (*n* = 2; replicates = 2).

% inhibition = 100 − (mean value of treated condition/mean value of untreated condition) × 100(2)

### 4.7. Evaluation of MMP-1 Levels in HaCaT Cells

The anti-photoageing activity was evaluated by investigating the product’s ability to reduce matrix metalloproteinase-1 (MMP-1) production in HaCaT cells after irradiation with UVB. For the quantification of the MMP-1 amount, an ELISA kit (MMP-1, Human, Biotrak ELISA System, Amersham, GE Healthcare, United Kingdom) was used. HaCaT cells were homogeneously seeded in 6-well plates and incubated at 37 °C in a 5% CO_2_ humidified atmosphere. After 24 h, the 2 highest concentrations (1.25 and 2.5 mg/mL), which were demonstrated to be non-cytotoxic after a preliminary MTT assay (data not shown) and with the best solubility were chosen to be tested in this assay. After the treatment time (24 h), cells were washed with Dulbecco phosphate buffer saline (DPBS, SIGMA-ALDRICH, St. Louis, MO, USA), irradiated with a single sub-toxic dose of UVB (5 mJ/cm^2^) and incubated at 37 °C with 5% CO_2_ for another 24 h. Untreated cells were used as control (Ctrl) and UVB-irradiated cells were used as a positive control, Ctrl (+). Subsequently, cell culture supernatants were collected according to the manufacturer’s instructions, and the concentration of MMP-1 in the samples was determined by interpolation from a 4PL standard curve (3.13–50 ng/mL) (*n* = 2; replicates = 2).

### 4.8. Evaluation of Skin Irritation on the Reconstructed Human Epidermis (RHE) 3D Model

The potential skin irritation of the enriched cream was evaluated in vitro on an RHE 3D model (SkinEthic, Nice, France), according to DB-ALM Protocol n°135 [20]. RHE inserts were placed in a maintenance medium (6-well plate) under sterile conditions and incubated at 37 °C, 5% CO_2_ overnight. After 24 h, the cream was applied in toto on the surface of the epithelium insert for 42 min at 32 μL/cm^2^ concentration.

Negative (DPBS) and positive controls, consisting of a 5% *w/v* solution of sodium dodecyl sulfate (SDS, Sigma-Aldrich, St. Louis, MO, USA) representing the irritating treatment, were also performed. At the end of the treatment, RHE inserts were rinsed with DPBS (25×) and then transferred into 6-well plates for incubation in a 37 °C, 5% CO_2_, 95% humidified atmosphere for 42 h. After this incubation time, the treated inserts were transferred into a 24-well plate filled with 300 µL of MTT solution (1 mg/mL), prepared according to the manufacturer’s instructions and incubated for a further 3 h at 37 °C, 5% CO_2_. Formazan crystals were dissolved in isopropanol (VWR Chemicals, Milan, Italy), and the OD of the samples was obtained by spectrophotometry at 570 nm wavelength using a microplate reader (*n* = 1; replicates = 3). The data were elaborated as a ratio of the corrected optical densities of the sample over the negative control (untreated sample), where cell viability values ≤50% are an index of irritation:

Cell viability (%) = [OD_570 nm_ test product/ OD_570 nm_ negative control] × 100 (3)

To better evaluate the skin irritation effect of the cosmetic cream, as well as the viability resulting from the direct contact of the product on the insert’s surface, the levels of interleukin (IL)-1α released after treatment were also measured after a recovery time of 42 h by an ELISA kit (Diaclone, Besançon cedex, France) following the manufacturer’s instructions. The absorbance was then measured at 450 nm using a microplate reader, and the IL-1α quantification was obtained by plotting the mean absorbance of each sample with a linear regression standard curve (3.9–250 pg/mL). IL-1α values ≥ 9 International Units (IU)/mL are considered an indicator of irritation.

### 4.9. Evaluation of Eye Irritation on Human Corneal Epithelium (HCE) 3D Model

The potential ocular irritation risk was screened on HCE inserts (Episkin Laboratories, Lyon, France) and in agreement with the reference standard method, DB-ALM Protocol n°190 [10]. After arrival, the inserts were placed in a 6 well-plate filled with the maintenance medium (Episkin Laboratories, Lyon, France) and incubated overnight in standard sterile condition (37 °C, 5% CO_2_ and 95% humidified atmosphere). After 24 h of equilibration, 30 µL of the cream in toto and 10 µL of DPBS were applied on the surface of the inserts. Then, 30 µL of DPBS was applied for the negative and 30 µL methyl acetate with 10 µL DPBS was applied for the positive control. Each condition was tested in triplicate. After 30 min of treatment, each insert was rinsed twice with DPBS and placed in a 24 well-plate containing the maintenance medium for 30 min of incubation in standard sterile conditions. After recovery time, all the inserts were transferred in a 24 well-plate prefilled with MTT 1 mg/mL and incubated for a further 3 h. Formazan crystals were extracted (4 h) in isopropanol, and then the absorbance was read at 570 nm with a microplate reader. The viability of tissue inserts was calculated as a ratio of the mean of each sample OD versus the mean of the negative control (inserts treated only with DPBS) OD (*n* = 1; replicates = 3).

### 4.10. Statistical Analysis

Results are presented as mean ± SD of at least three independent experiments performed in duplicate. Statistical significance was calculated using the one-way ANOVA followed by Fisher’s LSD test as post-hoc test. Values of *p* < 0.05 were considered statistically significant compared to the relative controls. Statistical analyses were performed using GraphPad Prism version 9.0.0 (GraphPad Software Inc, San Diego, CA, USA).

## Figures and Tables

**Table 1:** Functional classification of ingredients contained in the cream used in the study.

Function	Ingredients
Humectant	Propanediol, Glycerin, Caprylyl Glycol, Acetyl Heptapeptide-4
Emollient	Dimethicone, Cholesterol, Ethylhexylglycerin, Caprylyl Glycol, Glyceryl Caprylate, Glycine Soja Oil, Glycerin
Emulsifier	Carbomer, Sodium lauroyl lactylate, Xanthan gum, Sorbitan Stearate, Sorbityl 1-laurate, Polysorbate 20, Glyceryl Caprylate, Sodium oleate, Hydrogenated Lecithin, Cholesterol
Solvent	Aqua, Ethanol
Antimicrobic	Ethanol
Skin conditioning	Ceramide 3, Phytosphingosine, Ceramide 6 II, Ceramide 1, Caprylic/Capric Triglyceride, Butyrospermum Parkii Butter, Glycyrrhizic acid, *Centella asiatica* Leaf Extract, Acetyl Heptapeptide-4, Heptapeptide-10, Glycine Soja Oil, Dimethicone, Xanthan gum
Preservative & Perfume	Alcohol, Phenoxyethanol, Caprylic/Capric Triglyceride
Chelating Agent	Disodium EDTA
Surfactant	Sorbityl Laurate, Polysorbate 20, Sodium Oleate
Film Forming	Polyisobutene

## Data Availability

Data are included in the text; raw data are available from the corresponding author.

## Sample Availability

Samples of the analized compound (anti-ageing cream) is available from the authors.
